# Effectiveness of Electronic Flashcards for Undergraduate Medical Students in Musculoskeletal Sciences

**DOI:** 10.7759/cureus.77312

**Published:** 2025-01-12

**Authors:** Edward Laurent, Staton Phillips

**Affiliations:** 1 Trauma and Orthopaedics, Royal National Orthopaedic Hospital, Basildon, GBR; 2 Trauma and Orthopaedics, Mid and South Essex NHS Trust, Basildon, GBR

**Keywords:** active recall, anki, flashcard, online medical education, spaced repetition

## Abstract

Background

Electronic flashcards, such as Anki (https://apps.ankiweb.net/), are an educational resource that has been positively received by medical students worldwide. This is evidenced by surveys and improved examination results. However, little research has been conducted on the use of flashcards by undergraduate medical students at universities in the United Kingdom (UK), particularly within musculoskeletal sciences (MSK).

Research Question

The research question was: Are electronic flashcards (such as Anki) an effective educational tool for undergraduate medical students studying MSK?

Methods

Population

The study population included all medical students enrolled in the undergraduate course at Anglia Ruskin University (ARU), UK, during their MSK module rotation in 2023/2024.

Intervention

Each student was assigned a learning objective from the MSK curriculum and required to create Anki flashcards based on their assigned topic. These cards were then collated, quality-controlled, and redistributed to the entire cohort. This process not only created a comprehensive educational tool for the MSK syllabus but also introduced Anki to all students.

Outcome

The outcome was a survey employing a quantitative methodology using Likert scales (1 = least, 5 = most).

Results

The sample size was 61 students. The survey revealed that the effectiveness of electronic flashcards for studying MSK received a mean score of 4.03 (SD ± 1.05), with 50.1% of students rating it 5/5 on the Likert scale. Comparatively, the effectiveness of Anki in non-MSK modules showed a significantly lower mean score of 3.71 (SD ± 1.44), (*p* < 0.05).

The assignment also resulted in a significant increase in Anki usage, with the mean score rising from 3.34 (SD ± 1.48) before the intervention to 3.89 (SD ± 1.20) after (*p* < 0.05). Students who were new to Anki (n = 25) rated the effectiveness of the assignment in introducing them to the platform at a mean score of 3.98.

Conclusion

This survey demonstrated that electronic flashcards are perceived as an effective educational tool by undergraduate medical students in the UK. Flashcards were found to be more effective for studying within the MSK module compared to other modules. Furthermore, the assignment significantly increased participation in Anki by introducing students to a novel methodology for studying.

## Introduction

Undergraduate medical education requires students to learn and retain large quantities of information within a limited amount of time. This knowledge is not only required to pass the necessary examinations in the short term but is also required in the long term as doctors.

Conditions affecting the musculoskeletal (MSK) system account for 40% of consultations in primary care and an estimated 3.5 million emergency department (ED) attendances each year in the United Kingdom (UK) [1-3]. A systematic review conducted by Harkins et al. in 2023 revealed that both the quality of knowledge and confidence in undergraduate MSK students required improvement [4]. This highlights the importance of aiding medical students with additional educational tools [5,6].

Flashcards have long been recognised as a valuable educational tool in medical education. Classically, they are designed so that one side of the card displays ‘the topic' or 'question’, and the other side shows ‘the feedback' or 'answer’ [6]. It requires the student to actively retrieve knowledge, rather than simply passively absorbing. This is active recall, a proven high-utility study technique [7]. Augustin succinctly stated that long-term acquisition of knowledge can be broken down into three main components: elaboration, testing, and spacing [8]. The use of flashcards focuses on the testing and spacing components.

Flashcards have been digitalised through the use of various computer applications that create electronic flashcards (e-flashcards). Anki is an example, which is open-sourced and accessible from a computer or smart device [9].

Current literature and research themes

The existing literature on e-flashcards in medical education highlights several key themes, including the geographic location of studies and the level of students' medical education.

Research that was conducted in the United States investigated the effectiveness of Anki. Papers by Deng in 2015 and later by Lu in 2021 showed a positive correlation between the use of Anki and the results on the United States Medical Licensing Exams (USMLE) [10,11]. In the United States, Levy in 2023 noted that students performed significantly better in anatomical and physiology exams when using Anki at a higher level compared to light users (p<0.05) [12]. Survey-based studies in the United States and Australia demonstrated a high student receptivity and satisfaction with the use of Anki as an educational tool [12-14].

A key research gap identified was the limited literature on the use of Anki from UK-based medical schools. Furthermore, in the United States, students are required to have completed a pre-medical undergraduate degree prior to medical school. This makes them graduate-level students and further along in their educational journey when compared to UK medical schools, where this is not a requirement. Most of the literature was from the United States, but a paper focusing on undergraduate medical students was conducted by Jape et al. in 2022. This paper, conducted in Australia, looked at the effectiveness of Anki when studying pharmacology and found that Anki was ‘highly regarded’, with a positive global rating of 3.8 out of 5 on the Likert scale [14].

A further theme identified from the literature review was the specific medical subjects researched. Anki has been shown to be an effective educational tool in terms of student satisfaction when studying pharmacology, obstetrics and gynaecology, molecular biology, and psychiatry [13-15]. However, there is little research into Anki within the MSK subject. A subsection of MSK is anatomy. Levy in 2023 showed a positive student reception towards Anki when studying anatomy, with high Anki users giving a mean of 4.58 out of 5, which was considered significant [12]. However, this research focused on basic anatomy, while the MSK curriculum reflects more extensive clinical knowledge.

The conclusion of the literature review identified a research gap when assessing how UK-based medical undergraduate students perceive Anki flashcards while studying MSK.

## Materials and methods

Research question

Is the use of electronic flashcards (such as Anki) an effective educational tool for undergraduate medical students studying MSK?

Study design and selection

This was a primary research project with a study designed via the framework PIO (population, intervention, outcome) [16].

Population

The target sample was medical students in the undergraduate course at Anglia Ruskin University (ARU), UK. These students were in their second year and had completed a rotation in the MSK module for the year 2023/2024. The inclusion and exclusion criteria, as shown in Table 1, specify that all students from the second-year cohort were included, with the only exclusion being students who had interrupted their studies. Students who had taken time away from the medical course did not participate in the assignment.

**Table 1:** Inclusion and exclusion criteria Sample selection required students to meet the inclusion criteria outlined above.

Inclusion	Exclusion
Students enrolled in the undergraduate medical programme	Students who are intermitting (i.e. not active)
Students from Anglia Ruskin University	
Student studying in the MSK module in 2023/2024	

Intervention

The intervention involved the use of the electronic flashcard tool, Anki (https://apps.ankiweb.net/). Prior to this module, a proportion of students had already been using Anki as an educational tool. However, to gain a more accurate representation of this student cohort, it was important to introduce and educate students who were new to using Anki. This led to the creation of the Anki assignment.

The Anki Assignment

**Figure 1:**
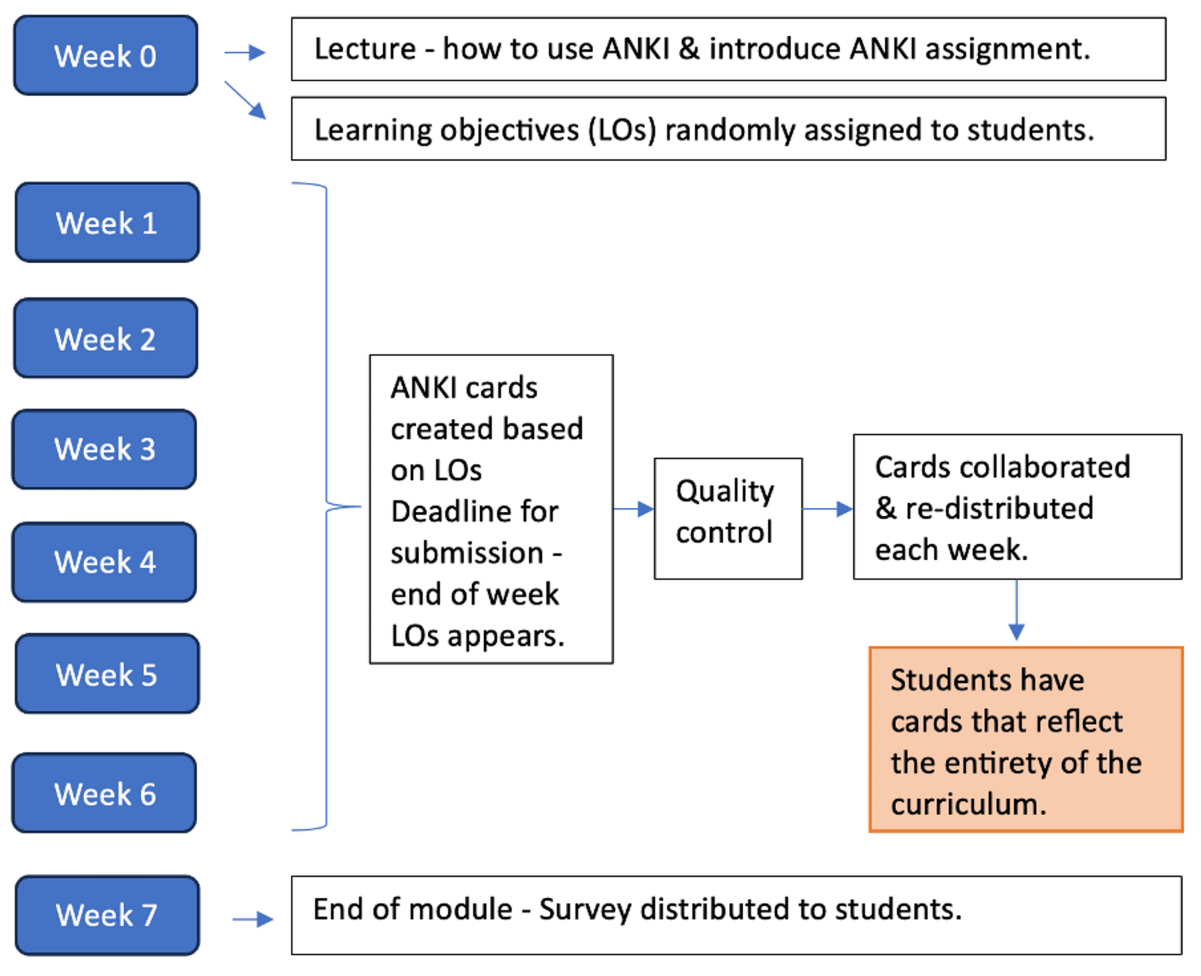
Timeline illustration of the Anki assignment This figure illustrated the sequence of events for the Anki assignment. Week 0 was at the start of the module. Week 1 - 6 was when all learning activities occurred. Week 7 was at the end of the module and when the survey was distributed. The musculoskeletal science block lasts 6 weeks, beginning with the introduction lecture and learning objective allocation. The deadline for submission of the Anki cards is the end of the week (Week 1 to 6) when that learning objective occurred. Once cards were received, they were quality-checked and re-distributed to the students. At the end of the assignment, the survey was distributed.

Each learning event within the MSK module at ARU is aligned with specific learning objectives. These events include lectures, anatomy dissections, workshops, and seminars. Students were randomly assigned a learning objective and tasked with creating three to five flashcards using Anki. The timeline is illustrated in Figure 1, with the deadline for submission set for the Sunday following the presentation of that learning objective.

At the start of the module, a teaching session was conducted to explain the assignment and included a tutorial on how to use Anki. This lecture was recorded and made accessible to all students for review. Additionally, a point of contact was established for any questions.

At the end of each week, the flashcards were collated and quality-controlled to ensure the information was factually accurate. The curated cards were then redistributed to the entire student cohort. By the end of the six-week module, students had received a complete set of flashcards covering the entirety of the curriculum.

The Anki assignment was mandatory and enforced by the head of the MSK module at ARU. Making the assessment voluntary would likely have resulted in poor participation, and the success of the project relied on all learning objectives having their corresponding cards. In cases where students did not create the required cards, these were completed by the assignment supervisor to ensure no learning objectives were missed.

Outcomes

The primary outcome was the students' perceived effectiveness of Anki in learning the MSK content. The secondary outcome was whether the assignment was an effective project to introduce or increase Anki's use.

Quantitative analysis was used as the methodology to evaluate the outcomes in the form of a survey. 

Survey

The survey was created using the platform ‘Online Surveys’ by the company Jisc (Bristol, UK), a trusted and ethically approved platform at ARU. A Likert scale (1 to 5) scoring system was employed, where 1 = least and 5 = most. For questions related to the frequency of flashcard use, additional descriptions were provided for the Likert scale: 1 = never, 2 = rarely, 3 = occasionally, 4 = often, and 5 = always.

The survey consisted of nine questions plus a participation consent question. All questions were scored using the Likert scale.

Results were interpreted through descriptive analysis of frequency and central tendency, including mean and median averages. Dispersion was measured using standard deviation, and upper and lower quartiles on the box plots. A paired Student t-test was used to identify any significant differences where the data was continuous and normally distributed. The results were presented in tables and box plots.

Ethical approval

The survey was anonymous. It was distributed with a participation information sheet, which outlined the research project. Students also provided consent for the use of their answers for research prior to being able to submit the survey. Ethical approval was granted by the School Research Ethic Panel (SREP) under the terms of ARU’s research policy (www.aru.ac.uk/researchethics). The ethics application number is ETH2223-8674.

## Results

Sixty-one students met the inclusion criteria and completed the survey. The median age was 20 years, and 57 (92%) students were undergraduates. At the end of week 6 the total number of cards created was 400. 

**Figure 2:**
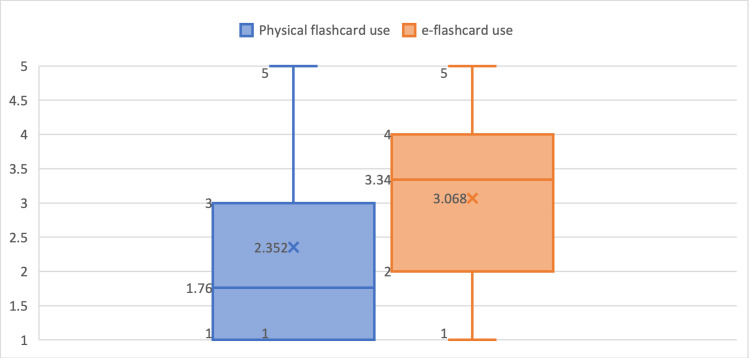
Box plot for medical students' use of physical and electronic flashcards This figure shows a box plot illustrating the significant increase in electronic flashcards (e-flashcards) use compared to physical flashcards.

Table 2 and Figure 2 show the students' usage of both physical and e-flashcards prior to the introduction of the Anki assignment. A paired Student’s t-test was used to statistically compare the usage of physical and e-flashcards. The result was a p-value of <0.05 - a significant increase.

**Table 2:** Results of the survey related to the usage of physical and electronic flashcards Table 2 illustrates the students' usage of both physical and electronic flashcards before the Anki assignment. It shows a significant increase in the use of electronic over physical flashcards with a Student's t-test producing p<0.05.

Survey question	Mean	Standard Deviation	Significant difference (student t-test)
How often do you use physical flashcards as part of your medical education? (n=61)	1.76	1.1	p<0.05
Prior to the Anki assignment: How often do you use electronic flashcards, such as Anki, as part of your medical education? (n=61)	3.34	1.48	

Table 3 shows how effective students found e-flashcards for studying MSK and compared it to their effectiveness in other modules. A paired Student's t-test produced a p-value <0.05, which showed a significant improvement in effectiveness within MSK. 

**Table 3:** Results of the survey related to the effectiveness of electronic flashcards such as Anki Table 3 illustrates a significant improvement in the effectiveness of electronic flashcards as a tool for medical education for musculoskeletal sciences (MSK) when compared to other non-MSK modules. This was shown with a Student's t-test producing a p-value of <0.05.

Survey question	Mean	Standard Deviation	Significant difference (student t-test)
How effective do you find electronic flashcards, (such as Anki) for studying MSK? (n=61)	4.03	1.05	p<0.05
How effective would you find electronic flashcards, such as Anki, as a tool in medical education for other modules (not MSK)? (n=61)	3.71	1.44	

**Figure 3:**
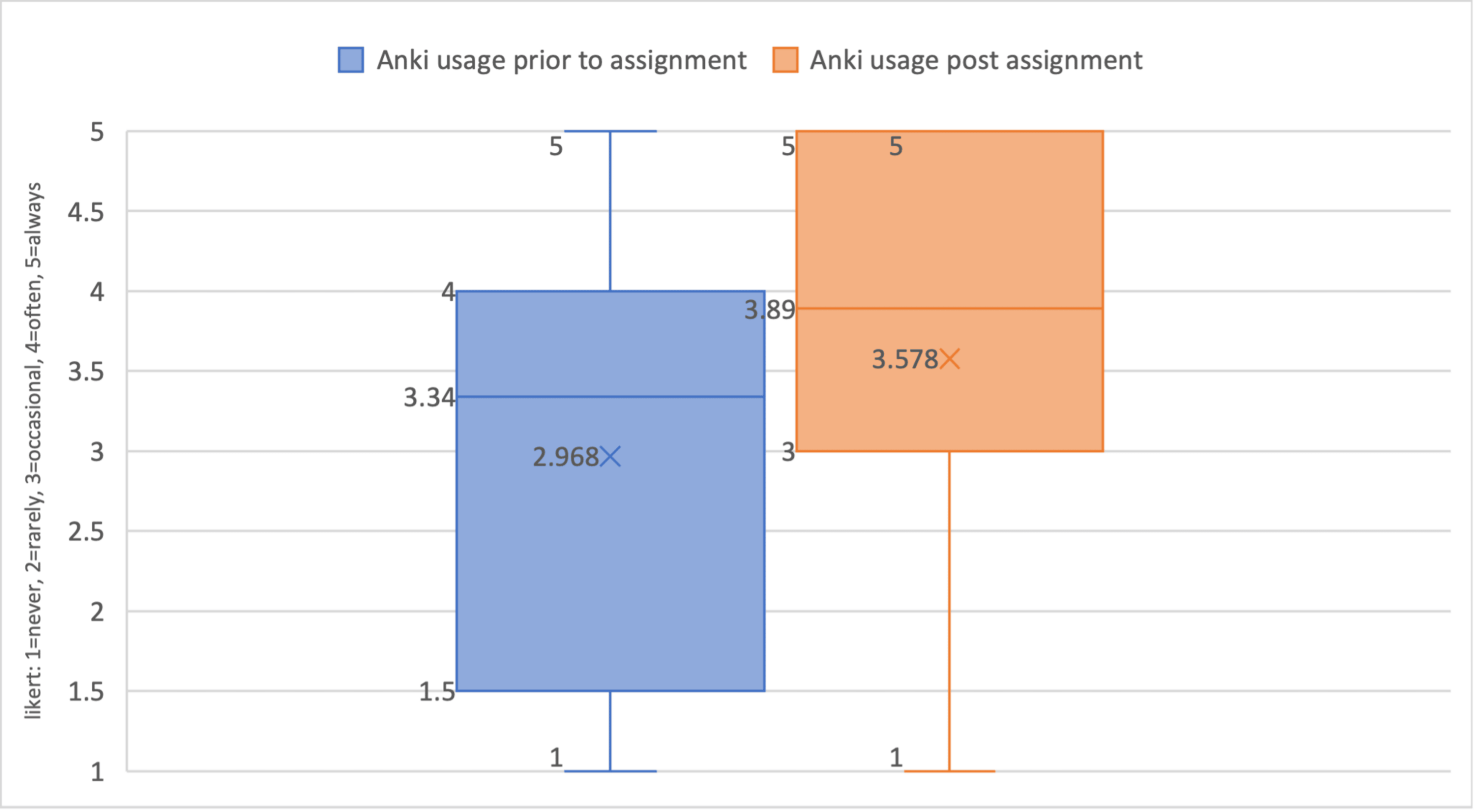
Box plot of Anki usage prior to the assignment and after A box plot that illustrates the comparison between the Anki usage prior to the assignment to the Anki usage after the assignment. It shows a clear increase in usage.

Table 4 illustrates the impact of the Anki assignment on participants' medical education within MSK. After the assignment, students used Anki significantly more, which is highlighted by a p<0.05 on the Student's t-test. Figure 3 illustrates this increase via a box plot. 

**Table 4:** Results of the survey related to the Anki assignment Table 4 illustrates the impact of the Anki assignment on students' medical education in terms of overall effectiveness and introducing a methodology to new users. There was also a statistically significant increase shown after the assignment, with a paired student t-test resulting in p<0.05.

Survey question	Mean	Standard Deviation	Significant difference (student t-test)
Prior to the Anki assignment: How often do you use electronic flashcards, such as Anki, as part of your medical education? (n=61)	3.34	1.48	p<0.05
After the Anki assignment: How often will you use electronic flashcards, such as Anki, for studying MSK? (n=61)	3.89	1.20	
How effective was this assignment (sharing the deck of cards of MSK syllabus) for your medical education? (n=61)	3.43	1.33	
If you were new to electronic flashcards, such as Anki, did this assignment help introduce a new method for your medical education? (n=25)	3.98	1.64	

## Discussion

Medical students are required to learn and retain large quantities of information in a short time. The MSK module alone comprises more than 150 different conditions [1]. Educational tools are essential to assist students with long-term knowledge acquisition, which is crucial for their careers as doctors.

Anki is an intervention that promotes revision and self-testing. It fosters active learning rather than passive absorption, which is fundamental for acquiring and retaining knowledge [8]. Anki has also been shown to be a more time-efficient method of revision [14,17]. Therefore, such strategies should be encouraged by educators.

Addressing the research question: the results indicate that undergraduate medical students from ARU find Anki to be an effective educational tool. These findings are consistent with currently published literature from the United States and Australia [11,13-15]. Anki was found to be significantly more effective for studying MSK compared to other non-MSK modules. This could be attributed to the anatomy and physiology content within MSK, areas where Anki has been shown to be popular with students [12].

The Anki assignment was designed to ensure all students were fairly exposed to the tool. A survey question that captured these students' experiences was: “If you were new to electronic flashcards, such as Anki, did this assignment help introduce a new method for your medical education?” The response was one of the highest scorers, with 19 (76%) rating it 4 or 5 out of 5 on the Likert scale. This high score reflected less on the assignment itself and more on the positive reception of Anki.

Anki was shown to be an effective educational tool because it utilises well-established educational theories. The first is the ‘testing effect,’ whereby the cards require self-assessment with immediate feedback. Compared to passive learning, the testing effect has been demonstrated to be superior in enhancing knowledge retention [8,18-20].

Additionally, Anki incorporates the ‘spacing effect,’ a concept first described by Ebbinghaus between 1880 and 1885. He found that long-term memory retention is more effective when learning events are spaced out over time, rather than occurring in immediate succession [21]. Since then, multiple studies have validated this theory, demonstrating its superiority for long-term knowledge acquisition by flattening the ‘forgetting curve’ [22-24]. Anki optimises this concept further by allowing students to self-rate the difficulty of the cards, which determines the time intervals for reviewing them. For instance, more challenging cards reappear sooner, whereas easier cards reappear after longer intervals. This feature enhances the time efficiency of revision, as students spend less time on concepts they have already mastered and more time on topics requiring additional focus [14].

This educational resource was created by students for students. A relatively small contribution (3-5 cards per student) resulted in a comprehensive set of 400 cards. Unique to this study was the creation of the cards by the students themselves, whereas other studies relied on pre-made cards [11,13,14,25]. Creating the cards encouraged students to reflect on their content before sharing it with peers. Such reflection is a habit that should be cultivated throughout their education, as it encourages critical evaluation of learned information and promotes higher-order learning [25]. The assignment also fostered a collaborative environment, where students worked together to support one another. Collaboration is a fundamental principle in their future careers as doctors and has been shown to positively impact the quality of patient care. This underscores the importance of developing these skills during medical training [26].

This study focused on students’ perceptions of the effectiveness of Anki cards. Using an exam-based approach, where Anki usage is correlated with performance, would be challenging due to the numerous variables involved, including the extent of other learning methods used. At best, the results would show an association rather than causation. The survey remains an appropriate methodology, as an educational resource can only produce outcomes if the students using it perceive it to be effective.

Limitations

A limitation of the assignment was the variation in the quality of the cards produced by the students. This was reflected in the mixed responses from the students, with the mean value for the question: “How effective was this assignment (sharing the deck of cards for the MSK syllabus) for your medical education?” being relatively low at 3.43. Several factors could have contributed to this outcome. Firstly, the assignment was formative rather than summative, with no grade or pass/fail outcome. The rationale for this approach was to reduce stress for students, with the expectation that the collective effort would yield high-quality work [27]. Despite the assignment being mandatory and a prize being awarded for the best cards, the extrinsic motivation provided was insufficient, relying too heavily on intrinsic motivation. While it is well documented that formative assessment can drive improved learning and outcomes [28], introducing summative assessment would require sufficient evidence of its effectiveness for medical students, which is one of the aims of this research paper. Another contributing factor was the robustness of the quality control procedures. The primary aim of these procedures was to ensure the accuracy of the information on the cards, not to amend or enhance their overall quality. A significant advantage of Anki as a digital platform, however, is its ability to allow users to easily edit, re-share, and delete flashcards, all of which contribute to potential improvements and future success [24].

Further research is needed to increase the sample size and to examine the use of Anki in different medical schools across the UK. Additionally, this study focused solely on Anki and did not consider other platforms that deliver e-flashcards. Comparing different software programs would also be beneficial, as this would allow educators to identify and promote the most effective tools.

## Conclusions

This survey addressed the primary research question, demonstrating that electronic flashcards were well received by undergraduate medical students from a UK university. The Anki flashcards were found to be more effective as an educational tool for studying MSK compared to other modules.

Overall, the Anki assignment successfully introduced a new educational methodology to medical students and encouraged those already familiar with it to utilise this resource more frequently. A low individual effort combined with peer collaboration resulted in the creation of a substantial resource to support their MSK education, both now and in the future.

The Anki assignment is an example of how educational institutions can harness technology in a formal way to improve medical education. 
